# Tyrosine Kinase Inhibitor Cabozantinib Inhibits Murine Renal Cancer by Activating Innate and Adaptive Immunity

**DOI:** 10.3389/fonc.2021.663517

**Published:** 2021-04-19

**Authors:** Hongyan Liu, Shishuo Sun, Gang Wang, Mengmeng Lu, Xiaokang Zhang, Xiaohuan Wei, Xiaoge Gao, Chao Huang, Zhen Li, Junnian Zheng, Qing Zhang

**Affiliations:** ^1^ Cancer Institute, Xuzhou Medical University, Xuzhou, China; ^2^ Center of Clinical Oncology, Affiliated Hospital of Xuzhou Medical University, Xuzhou, China

**Keywords:** cabozantinib, neutrophil, T cell, renal cell carcinoma, tumor microenvironment

## Abstract

**Background:**

Advanced renal cell carcinoma (RCC) has a very dismal prognosis. Cabozantinib, a tyrosine kinase inhibitor, has been approved for the treatment of advanced RCC. However, the impact of cabozantinib on the immune microenvironment of RCC remains poorly understood.

**Methods:**

Kaplan-Meier survival curves were constructed to examine the correlation between intratumor infiltration of neutrophils and patient prognosis in RCC. Infiltration and effector function of neutrophils and T cells in response to cabozantinib treatment were investigated in a murine RCC model.

**Results:**

A retrospective study of 307 RCC patients indicated that neutrophils were recruited into tumor tissues, and increased neutrophil infiltration was associated with improved clinical outcomes. In a murine model of RCC, cabozantinib treatment significantly increased both intratumor infiltration and anti-tumor function of neutrophils and T cells. Mechanistically, we found that cabozantinib treatment induced expression of neutrophil-related chemokines (CCL11 and CXCL12) and T cell-related chemokines (CCL8 and CX3CL1) in the tumor microenvironment. Furthermore, depletion of neutrophils and CD8^+^ T cells compromised the therapeutic efficacy of cabozantinib. Importantly, cabozantinib treatment induced long-term anti-tumor T cell response.

**Conclusions:**

Our study revealed novel mechanisms of the therapeutic effects of cabozantinib on RCC by activating both neutrophil-mediated innate immunity and T cell-mediated adaptive immunity. These findings are of great significance for guiding the clinical use of cabozantinib and provide a good candidate for future combination therapy with T-cell therapies or other immunotherapies.

## Introduction

Renal cell carcinoma (RCC), one of the most lethal urologic malignancies, is responsible for over 100,000 deaths every year with over 300,000 new patients diagnosed worldwide (1). The incidence of RCC continues to rise with a current rate of 2% per year worldwide. If identified at an early stage, radical surgical intervention offers a favorable prognosis, with the 5-year survival rate of 60% (2). However, approximately one-third of the RCC patients are diagnosed at advanced stages due to the asymptomatic nature of early disease stages (3). The landscape of treatment for advanced RCC has changed over the past decade with the introduction of targeted therapies. Currently, first-line treatment options include mTOR inhibitors and tyrosine kinase inhibitors combined with biological therapies, such as anti-VEGF drugs (4).

Cabozantinib, a receptor tyrosine kinase inhibitor, shows potent anti-tumor effects in animal and human studies through inhibition of receptor tyrosine kinase phosphorylation, particularly MET and VEGFR2 (5). In addition, cabozantinib treatment was shown to reduce tumor vascularity, increase autophagy and alter cell metabolism (6). In 2016, the U.S. Food and Drug Administration (FDA) approved cabozantinib for the treatment of advanced RCC based on data from one randomized, open-label, multicenter study (n=330), with a median progression-free survival (PFS) of 7.4 months and a median overall survival (OS) of 21.4 months compared to 3.8 months (PFS) and 16.5 months (OS) for everolimus (7).

Recently, numerous studies have indicated that in addition to their direct tumoricidal activity, some chemical drugs, such as doxorubicin (8, 9), sunitinib (10, 11) and sorafenib (12, 13), also exert their effects by modulating the tumor microenvironment and promoting anti-tumor immunity. Cabozantinib is approved for the treatment of advanced RCC; however, the impact of cabozantinib on the immune microenvironment of RCC remains poorly understood.

In the present study, we showed that intratumor infiltration of neutrophils was correlated with better prognosis in patients with RCC. In a murine model of RCC, cabozantinib treatment triggered infiltration of both antineoplastic neutrophils and T cells, and thus inhibited tumor growth. In addition, we demonstrated that adoptive transfer of cabozantinib-experienced T cells showed stronger antitumor efficacy *in vivo*. Our results reveal novel mechanisms of cabozantinib-mediated tumor growth inhibition by activating both innate and adaptive immunity.

## Materials and Methods

### Human RCC Samples and Neutrophil Staining

Tissue microarray (TMA) was run on 307 RCC tissues and 70 normal kidney tissues from patients who were enrolled at the Affiliated Hospital of Xuzhou Medical University from 2005 to 2008 in China. Clinicopathological and prognostic information from 247 patients were obtained from the medical records of the Affiliated Hospital of Xuzhou Medical University.

To detect tumor-infiltrating neutrophils, IHC staining was performed following a standard streptavidin-peroxidase (SP) method with the rabbit anti-human myeloperoxidase (MPO) monoclonal antibody (EPR4793, Abcam) at a dilution of 1:150 in PBS. Briefly, TMA slides were first deparaffinized, dehydrated and heat-induced epitope retrieval was performed in boiling citrate buffer (pH 6.0). Slides were subsequently blocked with goat serum and then stained with anti-MPO antibody at 4 °C overnight followed by addition of HRP-conjugated goat anti-rabbit antibody at room temperature. Next, the nucleus and cytoplasm were counterstained with hematoxylin & eosin. Stained slides were observed and imaged using a Nikon microscope. MPO-positive neutrophils were counted and analyzed. To analyses the relationship between infiltrated neutrophils and tumor progression/prognosis, the optimum cut-off value of infiltrating neutrophils was obtained by receiver-operator characteristic analysis.

### Cell Culture, Mice and In Vivo Treatment

Murine renal carcinoma Renca cells were cultured in complete RPMI-1640 medium supplemented with 10% FBS, 0.1 mM MEM nonessential amino acids (Cat# 11140-050, Gibco by Life Technologies), and 1 mM additional sodium pyruvate (Cat# 111360-070, Gibco by Life Technologies) at 37°C in a 5% CO_2_ atmosphere. Six- to eight-week-old female WT BALB/c, BALB/c Nude and NPG mice (NOD-Prkdc^scid^IL2rg^null^) were purchased from Beijing Vitalstar Biotechnology Company (Beijing, China) and housed in the specific pathogen-free animal facilities of the Experimental Animal Centre of Xuzhou Medical University. Mice were acclimated for approximately 1 week in our animal facility before experiments were initiated.

For the subcutaneous RCC model, Renca cells in RPMI media were injected into the right flank of mice. Mice were treated with cabozantinib (BMS-907351, XL184, Selleck) at a dose of 10 mg/kg in PBS administered by once-daily by oral gavage. For depletion of neutrophils or CD8^+^ T cells *in vivo*, anti-Ly6G-depleting antibody (1A8, BioXcell) or anti-CD8-depleting antibody (53.6.7, BioXcell) was administered to mice intraperitoneally at a dose of 200 μg/mouse every other day. During treatment, tumor length and width were measured using calipers, and tumor volume was calculated using the formula V = (L x W x W)/2, where V is tumor volume, W is tumor width, and L is tumor length. At the end of the treatment, blood, spleen and tumors were removed from mice. Then, tumor tissues were harvested, dissociated into a single-cell suspension, snap-frozen in liquid nitrogen or fixed in 4% paraformaldehyde.

### Flow Cytometry Analysis

For peripheral blood, red blood cells were lysed by hypotonic lysis using 1 X RBC Lysis Buffer (64010-00-100, PeproTech) in the dark at room temperature for 10 minutes. For the spleen, splenocytes were obtained by macerating spleens through a 70-μm nylon mesh. For tumors, tumor tissue was diced using ophthalmic scissors and then digested by incubating at 37°C for 1 hour in RPMI containing 10% FBS, 2 mg/mL collagenase IV, and 2 mg/mL DNase I. Single-cell suspensions of tumor tissue were obtained by straining through a 70-μm mesh filter, and cells were washed twice in PBS containing 2% FBS.

A total of 1x10^6^ cells were incubated with the indicated antibodies or isotype controls in staining buffer for 30 minutes at 4°C and then fixed in 1% paraformaldehyde. Fluorescence data were collected on a FACS machine (FACSCanto™ II, Becton-Dickinson, USA). Data analysis was performed using FlowJo 10 software (Ashland, OR). All antibodies and isotype controls were purchased from BD Biosciences, Biolegend or Affymetrix eBioscience. 7-Aminoactinomycin D (A1310) and Live/Dead Green dye (L23101) were purchased from Life Technologies.

### Flow Cytometry Sorting

Sample preparation was performed as described above. Single-cell suspensions were sorted immediately after labeling with the indicated antibodies conjugated with fluorochromes using a BD FACSAria™ III Cell Sorter, which incorporates 3 air-cooled lasers at 488-, 633-, and 407-nm wave lengths and is equipped with BD FACSDiva™ software. Sorted cells were incubated in complete SuperCulture™ L500 medium (Cat#: 6111021, Dakewe Bioengineering Co., Ltd) supplemented with 10% FBS and 30 U/ml IL-2.

### Wright-Giemsa Assay

CD11b^+^GR1^hi^ cells were sorted from Renca tumors that had received vehicle or cabozantinib treatment using a BD FACSAria™ III Cell Sorter. Next, cell concentrations were adjusted to 5 × 10^6^ cell/ml in 200 μl PBS, and cell smears were prepared using a cell smear centrifuge (Shanghai Lu Xiangyi Centrifuge Instrument Co., Ltd), spinning for 5 minutes at 2000 rpm. Then, the cell smear was placed on a dyeing rack according to the manufacturer’s protocol (Cat#: KAG227, Wright-Giemsa Assay Reagent), 3-5 drops of reagent 1 were added, and cells were stained for approximately 1 minute to fix the cell smear. Reagent 1 was not discarded, 6-10 drops of reagent 2 were directly added, the slide was gently shaken to mix the dye solution thoroughly and washed after 5-8 minutes, and then, cell morphology was assessed using an upright light microscope (Olympus Corporation, Tokyo, Japan).

### Hematoxylin and Eosin Staining and IHC

Tumor tissues were dissected, paraffin-embedded and sectioned (4 µm thickness). Sections were stained with hematoxylin and eosin (H&E), and immunohistochemistry was performed on formalin-fixed tumor tissue sections. To expose target proteins, heat-induced epitope retrieval (HIER) was performed in sodium citrate buffer (pH 6.0) for 20 minutes at 100°C. Tissues were blocked in 10% BSA for 20 minutes at room temperature, and anti-Ly6G antibody (RB6-8C5, Cat#: ab25377, Abcam) was applied to sections and incubated overnight at 4°C. After washing, colorimetric reactions were performed using two-step histostaining reagent kits (Beijing Zhong Shan Qiao Biotechnology Co., Ltd) according to the manufacturer’s protocol. The immunoreaction was visualized when brown precipitates formed following incubation in diaminobenzidine. Sections were subsequently washed with water and counterstained with 0.5% hematoxylin for 3 minutes at room temperature. Finally, tissues were visualized by an upright light microscope (Olympus Corporation, Tokyo, Japan).

### Real-Time Quantitative PCR

For gene expression analysis of murine Renca tumors, RNA was prepared from snap-frozen tissue using TRIzol (Cat#: R1100, Beijing Solarbio Science & Technology) according to the manufacturer’s protocol. Reverse transcription was conducted with 1 μg of total RNA using HiScript Q RT SuperMix for qPCR (Cat#: R123-01, Vazyme). Real-time quantitative PCR (qRT-PCR) analysis was performed using AceQ qPCR SYBR Green Master Mix (Cat#: Q111-02, Vazyme). A comparative CT (threshold cycle) was used to determine gene expression and analyzed against the endogenous murine β-actin gene. The following primers were used for murine CD3: 5′ATGCGGTGGAACACTTTCTGG3′, 5′GCACGTCAACTCTACACTGGT3′.

### Real-Time Cell Assay (RTCA)

The inhibitory effect of sorted neutrophils and T cells on proliferation in Renca cells was determined using a xCELLigence RTCA TP instrument (ACEA Biosciences). First, 50 μL of Renca cell culture media was added to each well of 2×8-well E-Plates (ACEA Biosciences), and the background impedance was measured and displayed as Cell Index. Then, Renca cells were seeded at a density of 10000 cells/well of the E-Plate in a volume of 100 μL and allowed to passively adhere onto the electrode surface. Post seeding, the E-Plate was kept at ambient temperature inside a laminar flow hood for 30 minutes and then transferred to the RTCAMP instrument inside a cell culture incubator. Data recording was initiated immediately at 15-minute intervals for the entire duration of the experiment. After approximately 10 hours, Renca cells reached a logarithmic growth phase, and the effector cells were added at different effector-to-target ratios in a volume of 50 μL. After transferring the E-Plate back into the xCELLigence system, data acquisition was resumed to monitor the neutrophil and T cell cytotoxic activity based on the viability of the attached target cells, as reflected by Cell Index values.

### IFN-γ Enzyme-Linked Immunospot Assay (ELISPOT)

To assess the ability of T cells sorted from splenocytes to generate IFN-γ, we used an ELISPOT assay (Cat#: 2210005, Dakewe Biotech Co., Ltd). Splenocytes were isolated from treated mice through 70-μm nylon wool, and T cells were purified using the EasySep Mouse T Cell Isolation Kit (Cat#: 19851, STEMCELL). PMA (500 ng/ml) and ionomycin (10 μg/ml) were added into each well to stimulate T cells. IFN-γ production and the formation of spots were evident following binding to antibody on the precoated plate.

### Adoptive T Cell Therapy

5×10^6^ Renca cells were subcutaneously inoculated into the right flank of BALB/c mice. Three days later, mice were randomly assigned into one of two groups (n=8-10/group) that received cabozantinib (10 mg/kg) or vehicle treatment once per day for 14 days. At the end of the treatment, T cells were sorted from the spleens of BALB/c mice using a BD FACSAria III Cell Sorter with anti-mCD45 and mCD3 antibodies staining. An s.c. mouse model of renal cancer was established in NPG mice using the Renca cell line (2×10^6^ cells/mouse). When tumors had grown to palpable size, tumor-bearing NPG mice were randomly assigned into one of two groups (n=5) and received 1×10^7^ sorted T cells from vehicle- or cabozantinib-treated mice through tail vein injection from one mouse to another mouse. During the experiment, tumor length and width were measured using calipers, and tumor volume was calculated as described above.

### Chemokine Antibody Array

Tumors treated with cabozantinib (10 mg/kg) or vehicle were excised and homogenized in PBS with protease inhibitor cocktail (Sigma) and 1% Triton X-100 (n=2). Concentrations of the extracted proteins were determined by both NanoDrop and a Pierce BCA protein assay kit (Thermo Scientific). Mouse Chemokine Antibody Array Kits (R&D Systems, ARY020) were used to detect relative expression levels of 25 chemokines. The analysis was performed according to the manufacturer’s protocol. Blots were developed using enhanced chemiluminescence, and images were obtained using a Fujifilm Imaging System (LAS-3000; Fujifilm, Tokyo, Japan).

### Statistics

GraphPad Prism 6 software was used for all statistical analyses. Results are presented as the mean ± SEM. Unpaired t-tests were used to assess significant differences between groups. Statistically significant *P*-values were labeled as follows: *P<0.05, **P<0.01, ***P<0.001, ****P<0.0001; ns, not significant.

## Results

### Increased Infiltration of Neutrophils Into the Tumor Bed Is Associated With Improved Clinical Outcomes in Patients With RCC

As key innate immune cells, neutrophils play a critical role in preventing pathogen invasion (14). Increasing evidence demonstrates that neutrophils are also involved in tumor progression (15). The neutrophil-lymphocyte ratio (NLR) in peripheral blood is a biological marker of inflammation with significant prognostic value in the field of oncology (16). A high NLR in peripheral blood upon diagnosis is significantly associated with an increased risk of recurrence and decreased OS and progression-free survival for several cancers, including RCC (17, 18). However, reports also indicate that neutrophil infiltration into the tumor may have beneficial effects. Tumor-infiltrating neutrophils prevent cancer progression by activating T cells as an antigen-presenting cell in early stages of lung cancer (19). Neutrophils were also shown to be recruited to lung by tumor-secreted chemokines to prevent metastasis of RCC through cytotoxic activity against metastatic cancer cells (20). However, the correlation between infiltrated neutrophils into the tumor bed and clinical outcome of RCC patients is still unclear.

**Figure 1:**
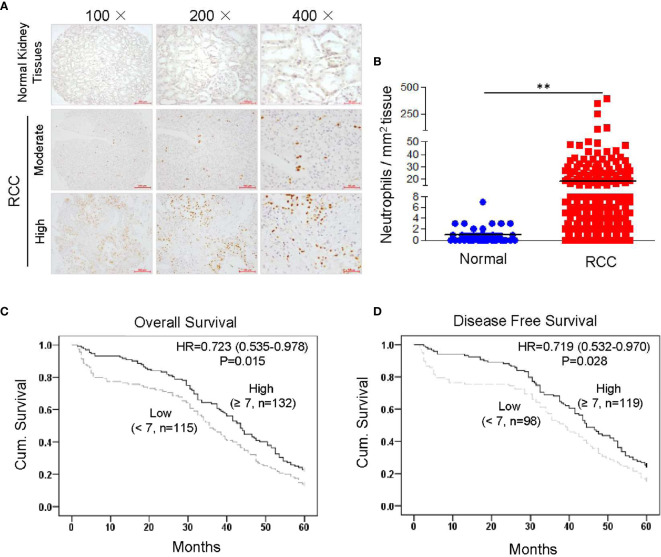
Higher levels of tumor-infiltrating neutrophils are correlated with improved clinical outcome in RCC patients. Tissue microarray slides, including 307 RCC tissues and 70 normal kidney tissues, were analyzed by IHC staining with anti-human MPO monoclonal antibody. MPO-positive neutrophils were counted and analyzed. The correlation between infiltrated neutrophils and prognosis was analyzed by constructing Kaplan-Meier survival curves with collected clinical information (*n* = 247, follow-up time = 60 months). Data are shown as the mean values ± SEM. **P<0.01. **(A)** Representative IHC staining results showing infiltrated neutrophils in RCC tissues and normal kidney. **(B)** Quantitative analysis of infiltrated neutrophils in normal kidney or RCC tissues (cell counts in 1 mm^2^ tissue). **(C)** High neutrophil infiltration correlated with better 5-year OS in 247 renal cell carcinoma patients (*p* = 0.015, log-rank test). **(D)** High neutrophil infiltration correlated with better 5-year disease-free survival in 217 renal cell carcinoma patients (*p* = 0.028, log-rank test).

We conducted a TMA assay using 307 RCC tissues and 70 normal kidney tissues collected from patients who were enrolled at the Affiliated Hospital of Xuzhou Medical University from 2005 to 2008 in China. Infiltrated neutrophils were identified by immunostaining of human MPO, which is the peroxidase enzyme most abundantly expressed in neutrophil granulocytes (19, 21). MPO-positive cells showed clear neutrophil-like segmented-nuclei (**Supplementary Figure 1**). TMA assay demonstrated that the number of infiltrated neutrophils was significantly increased in RCC tissues compared to normal kidney tissues (**Figures 1A, B**). To further clarify the correlation between infiltrated neutrophils and prognosis, Kaplan-Meier survival curves were constructed using collected clinical information (*n* = 247, follow-up time = 60 months). Results showed that increased neutrophil infiltration was positively associated with 5-year OS and disease-free survival in RCC patients (**Figures 1C, D**). Next, we assessed the relationship between infiltrating neutrophils and other clinicopathological features. No correlation was found between infiltrating neutrophils and age, gender, TNM (tumor, node and metastasis) stage or metastasis, etc. (**Supplementary Table 1**). These data demonstrated that increased neutrophil infiltration into the tumor bed was associated with improved clinical outcome in RCC patients.

To further confirm whether infiltrating neutrophils represent an independent prognostic marker for RCC, univariate and multivariate Cox regression analyses were performed. Results revealed that number of infiltrating neutrophils was an independent prognostic marker for both OS and disease-free survival in RCC patients (**Supplementary Tables 2**, **3**). The data indicated that increased numbers of tumor infiltrating neutrophils represent a marker for predicting survival in RCC patients.

### Cabozantinib Treatment Leads to Increased Neutrophil Infiltration Into the Tumor Microenvironment and Growth Inhibition of Murine RCC

**Figure 2:**
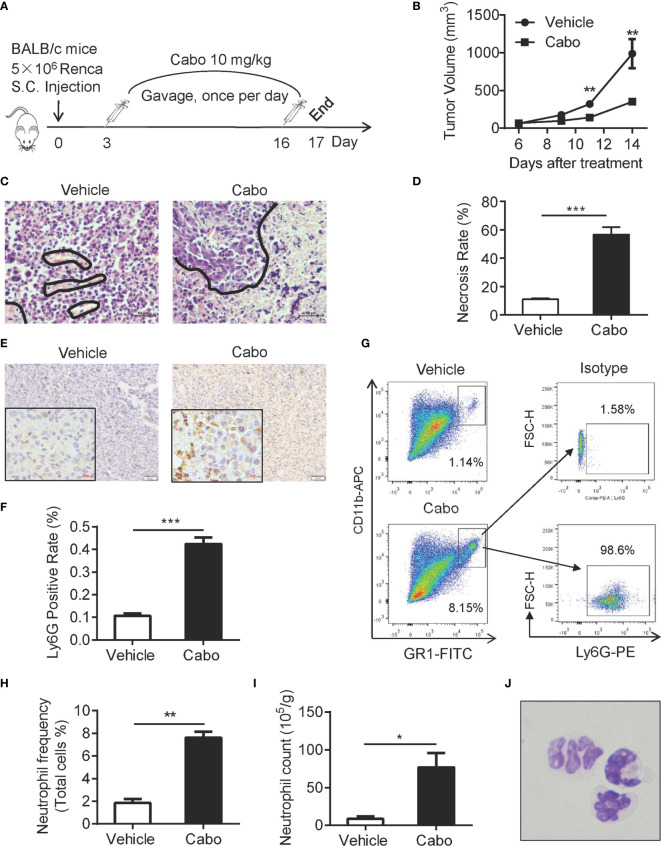
Cabozantinib causes tumor growth inhibition and neutrophil infiltration into the tumor bed in a murine model of RCC. **(A)** Schematic diagram of the experimental design. 5×10^6^ Renca cells were subcutaneously inoculated into the right flank of BALB/c mice (day 0). On day 3, mice were randomly divided into 2 groups that received cabozantinib (10 mg/kg) or vehicle treatment once per day for 14 days. **(B)** Tumor growth curves during the experiment. **(C, D)** Representative results of H&E staining and quantitative analysis of necrotic area in tumor tissues. The area encircled by blue coil indicates necrotic tissue. **(E, F)** Representative IHC staining (anti-Ly6G, Brown color) and quantitative analysis of Ly6G^+^ cells in tumor tissues. **(G)** Tumor tissue was homogenized into single cell suspension. 1X10^6^ cells were stained with anti-mouse CD45, CD11b and Gr1 and ly6G antibodies, and analyzed by FACS. Representative FACS plots showing the frequencies of CD11b^+^Gr1^hi^ cells in cabozantinib- or vehicle-treated tumors (left) and expression of Ly6G on CD11b^+^Gr1^hi^ cells. **(H)** Quantitative analysis of CD11b^+^Gr1^hi^ cell frequencies in tumors that received cabozantinib or vehicle treatment. **(I)** Quantitative analysis results of CD11b^+^Gr1^hi^ cell count per gram of tumor tissue. **(J)** Morphological identification of CD11b^+^Gr1^hi^ cells by Wright-Giemsa staining. Images were taken under 600× magnification. Representative data (Vehicle, n=8; Cabozantinib, n=10) of three independent experiments are shown (Vehicle, n=24; Cabozantinib, n=28 in total) with mean values ± SEM. *P<0.05, ***P<0.001 by unpaired t-test.

A previous report showed that cabozantinib triggers neutrophil-mediated anti-tumor effects in a mouse model of prostate cancer (22). Based on our observation that increased neutrophil infiltration into tumors in RCC patients was associated with better clinical outcome (**Figure 1**), we investigated whether cabozantinib treatment enhances the infiltration and anti-tumor function of neutrophils in a murine model of RCC. The proliferation inhibitory effect of cabozantinib in the murine RCC Renca cell line was confirmed *in vitro* (**Supplemental Figure 2**). We established a murine model of RCC by subcutaneous (s.c.) injection of Renca cells into BALB/c mice (**Figure 2A**). We found that cabozantinib treatment significant inhibited RCC tumor growth (**Figure 2B**). Histopathologic evaluation of Renca tumors from treated mice indicated that cabozantinib treatment induced large amounts of tumor tissue necrosis (**Figures 2C, D**), which was accompanied by increased Ly6G^+^ cell infiltration (**Figures 2E, F**). Flow cytometry analysis also showed increased CD11b^+^Gr1^hi^ populations in tumors after cabozantinib treatment (**Figures 2G–I**). Further analysis revealed that almost all CD11b^+^Gr1^hi^ cells were Ly6G positive neutrophils (**Figure 2G**). Morphological identification of the CD11b^+^Gr1^hi^ cells isolated from tumors by Wright-Giemsa staining confirmed that the CD11b^+^GR1^hi^ cells were neutrophils (**Figure 2J**).

### Neutrophils Are Required for Cabozantinib-Mediated Tumor Regression

**Figure 3:**
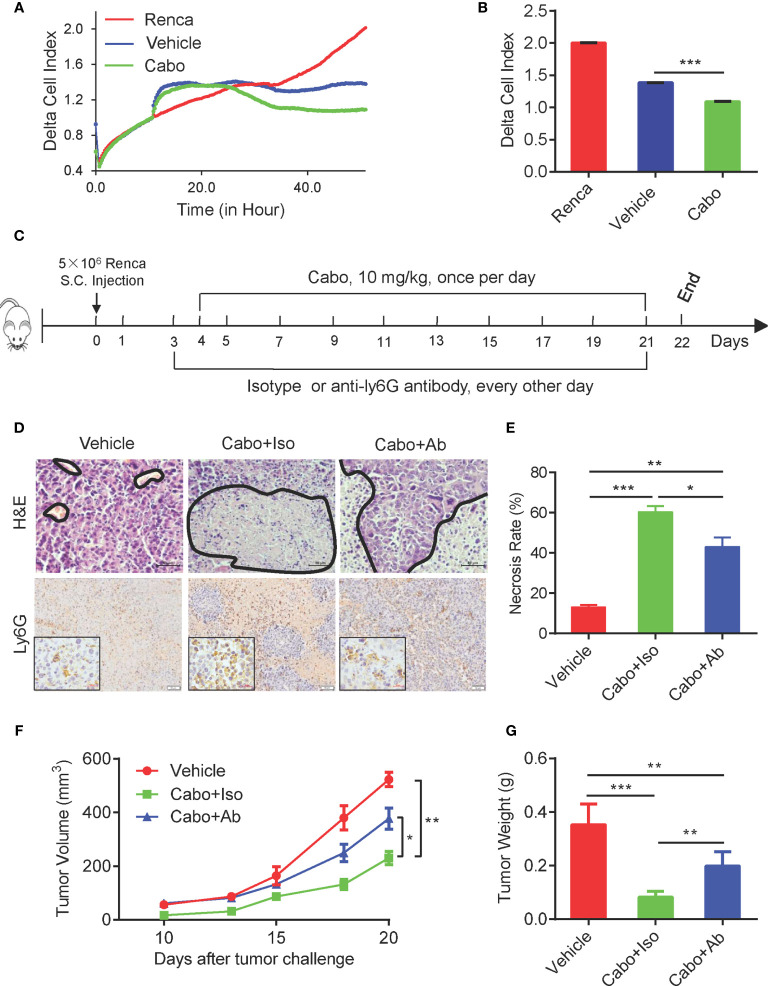
The therapeutic effect of Cabozantinib is partially dependent on neutrophils. **(A)** Tumor growth inhibition effect of cabozantinib-primed neutrophils by RTCA. Mice were treated as in **Figure 2A**. At the end of the experiment, neutrophils were sorted from tumor tissues of vehicle and cabozantinib-treated mice for the *ex vivo* functional assay. Renca cells (1x10^4^ cells/well) were seeded into the E-Plate of RTCA machine. When cells reached a logarithmic growth phase (approximately 10 hours), neutrophils were added into the E-Plate at an E:T ratio = 5:1. Proliferation of Renca cells was monitored in real time by RTCA (n=3 for biological replicate, and n=2 for technique replicate). **(B)** Statistical analysis of the cell index at the end of RTCA (50 hours). For A and B, experiments were repeated three times. **(C)** Schematic diagram showing treatments of mice. BALB/C mice were subcutaneous inoculated with Renca cells. Mice were then randomly assigned to 3 groups (n=6 mice per group): vehicle, cabozantinib + isotype control and cabozantinib + anti-Ly6G-depleting antibody at day 3 and anti-Ly6G-depleting antibody or its isotype control antibody (200 µg/mouse) was administered i.p. every other day. Cabozantinib (10 mg/kg) or vehicle was administered by gavage once per day, beginning on day 4. **(D, E)** Representative H&E (upper panel, the area encircled by the blue coil indicates necrotic tissue), and IHC (lower panel) staining results of tumor tissues, and quantitative analysis of necrotic area in tumor tissues. **(F)** Tumor growth curves during the experiment. **(G)** The average tumor weight of each group at the end of the experiments. Representative data of three independent experiments are shown (Vehicle, n=18; cabozantinib + isotype, n=18; cabozantinib + anti-Ly6G-depleting antibody, n=18 in total) with mean values ± SEM. *P<0.05, **P<0.01, ***P<0.001 by unpaired t-test.

Recent reports have shown that neutrophils exert direct cytotoxicity against tumor cells (23, 24). Their cytotoxicity requires direct physical interaction with tumor cells and is mediated by hydrogen peroxide (H_2_O_2_) released by neutrophils and transient receptor potential cation channel (TRPM2), an H_2_O_2_-dependent Ca^2+^ channel expressed on the surface of cancer cells (25, 26). To investigate whether neutrophils induced by cabozantinib exhibit anti-tumor activity, we sorted neutrophils from cabozantinib-treated Renca tumors and cocultured them with Renca cells *in vitro*. The direct proliferation inhibitory effect of neutrophils on Renca cells was analyzed using a real-time cell assay (RTCA). Results demonstrated that neutrophils sorted from both vehicle- and cabozantinib-treated tumors significantly inhibited the growth of Renca cells. However, neutrophils from cabozantinib-treated tumors exhibited much stronger inhibitory efficacy (**Figures 3A, B**).

The increased tumor infiltration and enhanced inhibition of tumor growth by tumor-infiltrating neutrophils after cabozantinib treatment suggested that neutrophils might play an important role in cabozantinib-mediated tumor growth inhibition. To test this possibility, BALB/c mice bearing Renca cell tumors were pretreated with anti-Ly6G-depleting antibody (1A8) to deplete neutrophils before cabozantinib treatment (**Figure 3C**). FACS analysis with anti mouse CD11b and GR1 (RB6-8C5) antibodies showed that Anti-Ly6G antibody treatment resulted in almost complete depletion of neutrophils in the peripheral blood (**Supplemental Figures 3A, B**). We adapted the FSC/SSC gating strategy to further confirm that neutrophils were almost completely depleted by Ly6G antibody in our study (**Supplemental Figure 3C**). The IHC results of tumor tissues also showed that the tumor-infiltrating neutrophils were significantly decreased in anti-Ly6G-depleted antibody treated mice (**Figure 3D** lower panel). The tumor growth curve showed that depletion of neutrophils attenuated cabozantinib-mediated anti-tumor efficacy (**Figures 3F, G**). Moreover, cabozantinib treatment-induced tumor tissue necrosis was also decreased in the absence of neutrophils during treatment (**Figure 3D** up panel, and **Figure 3E**). These data indicate that neutrophils are required for cabozantinib-induced tumor growth inhibition.

### Cabozantinib Treatment Upregulates Chemokines in the Tumor Microenvironment

**Figure 4:**
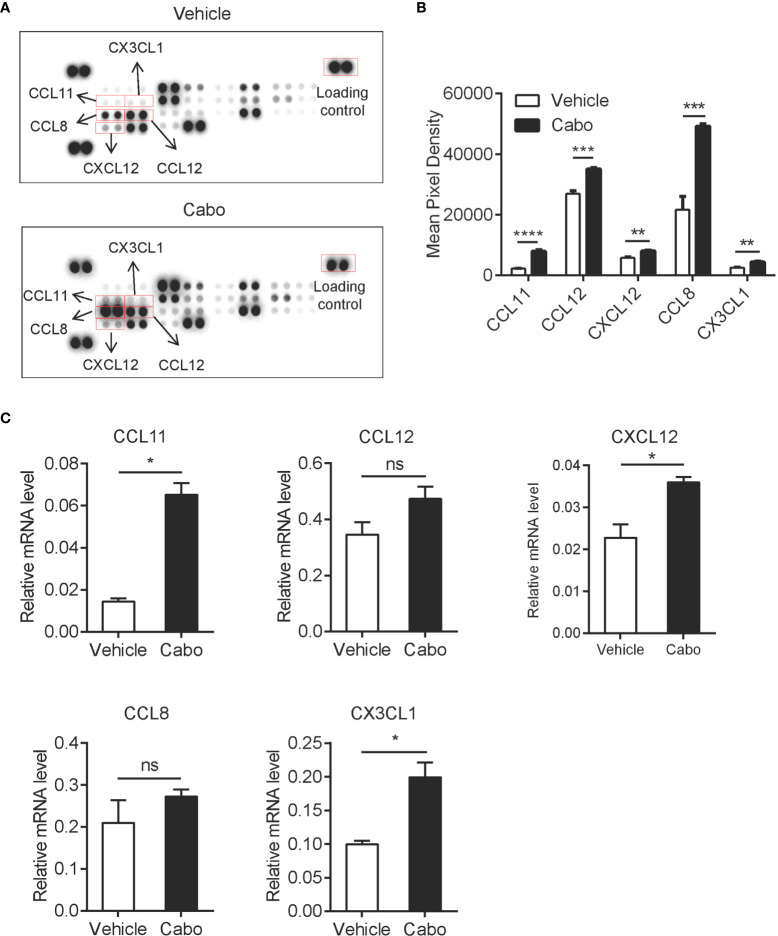
Cabozantinib causes upregulation of chemokines in the tumor microenvironment. Mice were treated as in **Figure 2A**. **(A, B)** At the end of the experiment, total protein was extracted from tumor tissues of two random mice per group. Levels of 25 chemokines were detected using mouse chemokine antibody array kits. Chemokine expression levels are presented as arbitrary units measured by densitometry (n=2, biological replicate). Representative films **(A)** and quantitative results **(B)** of chemokines by chemokine array analysis. **(C)** RNA was prepared from snap-frozen tumor tissue (n=3, biological replicate). Relative mRNA expression of chemokines was determined by qRT-PCR. Data are shown as the mean values ± SEM. *P<0.05, **P<0.01, ***P<0.001, ****P<0.0001 by unpaired t-test; ns, not significant.

Chemokines serve as critical players in immune cell migration (27). One of the possible reasons for the increased infiltration of neutrophils in the tumor microenvironment after cabozantinib treatment is the induction of chemokines. To confirm this hypothesis, we assessed expression levels of 25 chemokines in tumor tissues recovered from tumor bearing mice that had received cabozantinib or vehicle treatment using chemokine antibody array analysis (**Supplementary Table 4**). We found that cabozantinib treatment significantly upregulated CCL11, CCL12, CXCL12, CCL8 and CX3CL1 in the tumor microenvironment (**Figures 4A, B**). qPCR analysis also indicated that mRNA levels of all five chemokines in cabozantinib-treated tumors were higher than those in vehicle-treated tumors, though there was no significant difference for CCL12 or CCL8 (**Figure 4C**).

CCL11 has been shown to promote migration of neutrophils during human allergic reactions and in a murine lung injury model (28, 29). CCL12 specifically attracts monocytes (30). CXCL12 was reported to be upregulated by cabozantinib to promote infiltration of neutrophils into the tumor bed in murine prostate cancer (22). Data from our study and previous reports suggest that increased tumor infiltration of neutrophils induced by cabozantinib might be due to the increased neutrophil-related chemokines CCL11 and CXCL12 in the tumor microenvironment. Interestingly, we found that two T cell chemokines, CCL8 and CX3CL1, were also upregulated after cabozantinib treatment. CCL8 was reported to chemoattract T cells (31–33). We performed *in vitro* chemoattraction assays and demonstrated that CCL8 is an important chemokine for T cells migration (**Supplementary Figure 4**). CX3CL1 reportedly promotes T cell aggregation in tumor tissue and is positively correlated with patient prognosis (34–36). These results indicated that cabozantinib therapy may also trigger T cell infiltration to the tumor site.

### Cabozantinib Treatment Increases Infiltration of T Cells into the Tumor Microenvironment

**Figure 5:**
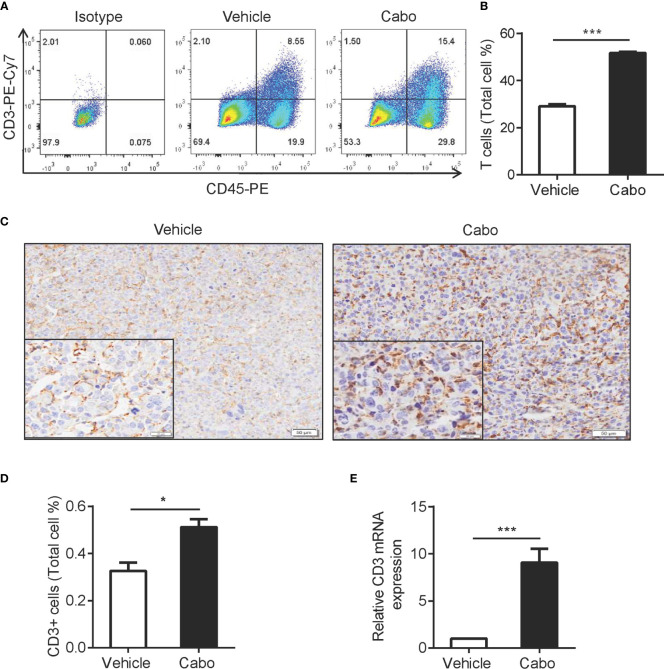
Cabozantinib treatment increases T cell infiltration into tumors. Mice were treated as in **Figure 2A**. **(A, B)** Representative FACS plot and quantitative results show the frequencies of T cells in tumors from vehicle- or cabozantinib-treated mice. **(C, D)** Representative IHC staining for CD3 **(C)** and quantitative results **(D)** of tumor infiltrating T cells in vehicle- or cabozantinib-treated mice. CD3 positive rates **(D)** were analyzed using Image-Pro Plus software based on 3 representative visual areas in each section from each tumor (n=6 per group). **A-D** experiments were repeated three times. **(E)** Mouse CD3 mRNA expression detected by quantitative RT-PCR. RNA was extracted from tumor tissue from mice that received vehicle or cabozantinib treatment. Expression levels are normalized to vehicle (n=3 for biological replicate). *P<0.05, ***P<0.001 by unpaired t-test. This experiment was repeated two times.

The adaptive immune response is reportedly involved in tumor regression mediated by tumor-targeted therapies, such as anti-HER2/neu antibody therapy and EGFR TKI (37, 38). The results above showed that cabozantinib treatment increased the expression of CCL8 and CX3CL1 in the tumor environment, which is important for T cell migration (**Figure 4**). We then wanted to determine whether T cells are also involved in cabozantinib-mediated tumor growth inhibition. We found that cabozantinib significantly increased the frequency of T cells in tumor tissue compared to vehicle as indicated by flow cytometry (**Figures 5A, B**), immunohistochemical staining (**Figures 5C, D**) and CD3 mRNA detection (**Figure 5E**). These data demonstrated that cabozantinib treatment results in increased infiltration of T cells into the tumor microenvironment.

### T Cells Are Required for the Anti-Tumor Efficacy of Cabozantinib

**Figure 6:**
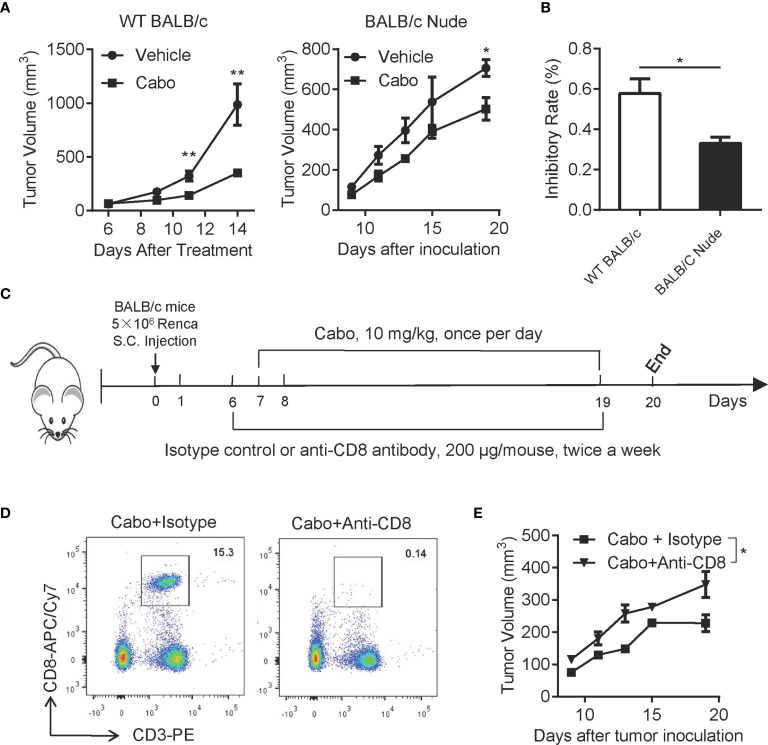
CD8^+^ T cells are required for cabozantinib-induced inhibition of tumor growth. **(A)** Tumor growth inhibition by cabozantinib in wild type (WT) BALB/c and BALB/c Nude mice. 5×10^6^ Renca cells were subcutaneously inoculated into the right flank of WT BALB/c and BALB/C Nude mice. Seven days after tumor inoculation, mice (n=5 per group) received either vehicle or cabozantinib (10 mg/kg, once per day by gavage) treatment for 13 days. **(B)** Tumor growth inhibition rates by cabozantinib treatment in WT BALB/c and BALB/c Nude mice. This experiment was repeated 3 times. The results of one representative experiment are shown. **(C)** Schematic diagram of the experimental design. Mice were subcutaneously inoculated with Renca cells (day 0). At day 6 (one day before cabozantinib treatment), mice were randomly assigned to 4 groups (n=6 for each group) and received antibodies treatment as indicated: vehicle + isotype, cabozantinib + isotype control, vehicle + anti-CD8-depleting antibody and cabozantinib + anti-CD8-depleting antibody. **(D)** Representative FACS plot showing CD8^+^ T cells in peripheral blood 24 hours after intraperitoneal injection of anti-CD8 depleting antibody. **(E)** The tumor growth curve of each group during treatment. This experiment **(C-E)** was repeated 2 times. The results of one representative experiment are shown. *P<0.05, **P<0.01, by unpaired t-test; ns, not significant.

We next investigated whether T cells were essential for cabozantinib-mediated anti-tumor effects. We treated both tumor-bearing wild type (WT) BALB/c mice and BALB/c Nude mice with cabozantinib and evaluated tumor growth inhibition in the two types of mice. As shown in **Figure 6A**, cabozantinib treatment in WT BALB/c mice resulted in significant tumor growth inhibition; however, cabozantinib had very limited tumor growth inhibitory effects in BALB/c Nude mice with T cell deficiency. The tumor growth inhibition rate of cabozantinib in WT BALB/c mice was significantly higher than in BALB/c Nude mice (**Figure 6B**). These data suggest that T cells might be involved in cabozantinib-mediated tumor growth inhibition.

CD8^+^ T cytotoxic lymphocytes are a major adaptive immune cell population involved in controlling tumor growth. To further confirm whether CD8^+^ T cell were essential for cabozantinib-mediated anti-tumor effects, BALB/c mice bearing Renca tumors were treated with anti-CD8-depleting antibody during cabozantinib treatment (**Figures 6C, D**). We found that cabozantinib-mediated tumor growth inhibition was significantly attenuated in response to CD8 depletion (**Figure 6E**). Together, these data indicate that T cell-mediated anti-tumor immunity plays an important role in cabozantinib-mediated tumor growth inhibition.

### Cabozantinib Treatment Enhances the Anti-Tumor Effects of T Cells

**Figure 7:**
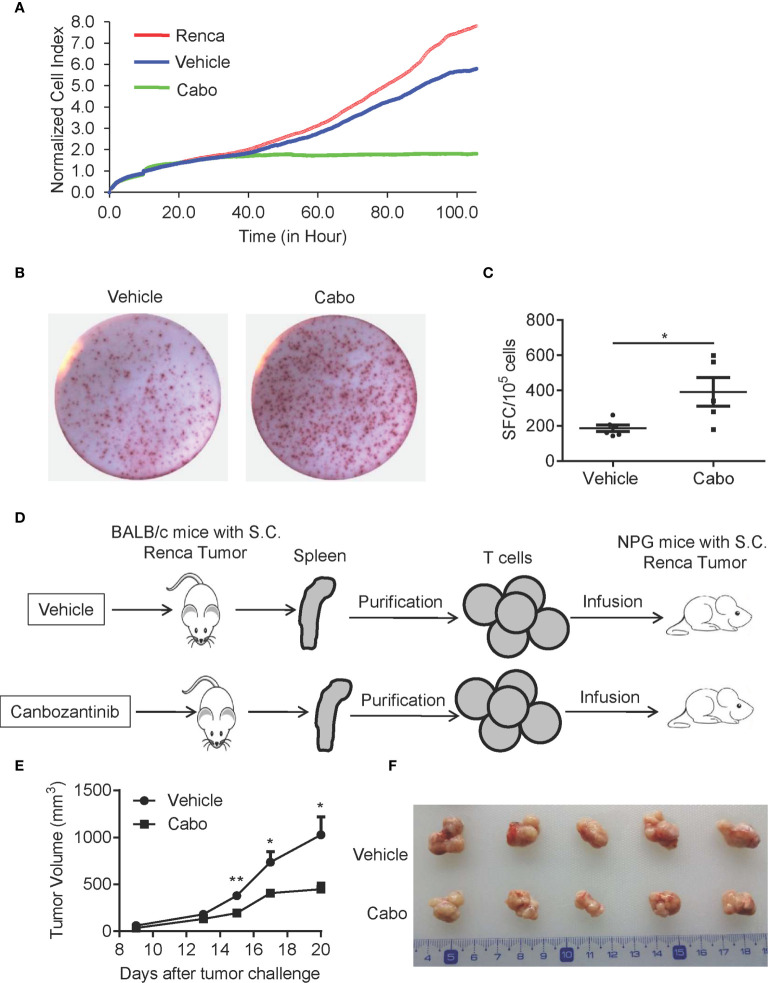
Cabozantinib treatment enhances the anti-tumor effects of T cells. **(A)** T cells were isolated from tumors of BALB/c mice that received vehicle or cabozantinib treatment as in **Figure 2A** and were cocultured with Renca cells at a ratio of E:T=2:1. The cytotoxicity of T cells was measured by RTCA (n=3 for biological replicate). **(B, C)** Representative plots and quantitative results of IFN-γ production by splenic T cells by ELISPOT assay. T cells were purified from splenocytes and were then stimulated with PMA (500 ng/ml) and ionomycin (10 μg/ml). IFN-γ spots were analyzed (n=3 for biological replicate). **(D)** Schematic diagram of the experimental design. T cells isolated from vehicle or cabozantinib treated tumor bearing BALB/c mice were adoptively transferred to NPG mice with palpable RCC tumors. **(E)** Tumor growth curves of NPG mice receiving T cells from different sources (n=5 per group). **(F)** Representative tumor images from different treatment groups. These experiments were repeated 2 times. *P<0.05, **P<0.01 by unpaired t-test.

Next, we further examined whether cabozantinib treatment enhances the anti-tumor activity of T cells. We sorted T cells from tumor tissue of vehicle- or cabozantinib-treated mice and cocultured them with Renca cells *in vitro*. The anti-tumor effect of T cells was analyzed by RTCA. As shown in **Figure 7A**, cabozantinib treatment resulted in significantly increased cytotoxicity of tumor-infiltrating T cells compared to vehicle treatment. To further confirm the effector function of T cells, T cells were purified from splenocytes of vehicle- or cabozantinib-treated Renca tumor-bearing mice, and IFN-γ production by T cells was assessed by ELISPOT assay. As shown in **Figures 7B, C**, IFN-γ production of T cells from cabozantinib-treated mice was significantly enhanced compared to T cells from vehicle-treated mice in response to PMA + ionomycin treatment *ex vivo*.

To further confirm the effects of cabozantinib treatment on T cell antitumor activity, we sorted T cells from splenocytes of vehicle- or cabozantinib-treated BALB/c mice with Renca tumors. Then, the purified T cells were infused into immunodeficient NPG mice with established s.c. Renca tumors (**Figure 7D**). As shown in **Figures 7E, F**, T cells from cabozantinib-treated BALB/c mice exerted stronger tumor growth inhibition than those from vehicle-treated mice. Together, these results demonstrated that cabozantinib treatment enhances the anti-tumor effect of T cells.

## Discussion

Cabozantinib, a receptor tyrosine kinase inhibitor, has shown potent anti-tumor effects in both animal and human studies (39, 40) and is approved for the treatment of advanced RCC. Although the concept of the anti-tumor activity of neutrophils and the effect of cabozantinib to induce anti-tumor neutrophils has been reported in murine prostate cancer model (22), however, whether the theory can be extended to other cancers and whether adaptive immunity is also involved in cabozantinib mediated tumor inhibition are not clear. Our current study had following novel findings: 1) we reported that tumor infiltration of neutrophils was positive correlation with clinical outcome in patients with RCC; 2) cabozantinib treatment induced anti-tumor neutrophils in murine RCC model; 3) importantly, our work demonstrated that CD8^+^ T cells also participated in the cabozantinib mediated anti-tumor process. Our study thus provided the first evidence that both the innate and adaptive immunity were involved in cabozantinib-mediated renal cancer therapy.

Neutrophils are a type of phagocyte that are normally found in the bloodstream. Several studies have indicated that elevated levels of neutrophils in the peripheral blood are closely associated with tumor progression and poor clinical outcome (41–43). In particular, the NLR and neutrophilia are independent prognostic markers in many types of cancers, including RCC (44–46). However, these findings are primarily based on the detection of NLR in peripheral blood. Distinct from NLR in the blood, the prognostic and predictive power of tumor-infiltrated neutrophils is variable. Elevated tumor-infiltrated neutrophils in patients with colorectal carcinoma were shown to be associated with improved survival and prognosis (47, 48). However, in patients with melanoma, intratumoral neutrophils were reportedly negatively correlated with patient outcome (49, 50). Here, we demonstrated that neutrophils were infiltrated in the tumor bed of patients with RCC. Importantly, increased neutrophil infiltration into the tumor bed was associated with better clinical outcome in patients with renal cell cancer. Moreover, the results of univariate and multivariate Cox regression analysis revealed that the number of infiltrated neutrophils was not affected by age, gender, tumor size, metastasis or TNM stage (**Supplementary Tables 3**, **4**).

In addition, in our study, we demonstrated that cabozantinib treatment enhances anti-tumor T cell immunity. We found that cabozantinib treatment results in increased T cell infiltration into the tumor bed. Furthermore, cabozantinib treatment significantly enhanced the cytotoxicity of tumor-infiltrated T cells and IFN-γ production of T cells from splenocytes. Increased levels of chemokines (CCL8 and CX3CL1) may contribute to the infiltration of T cells. Moreover, tumor antigens released from the necrotic tumor tissue caused by cabozantinib treatment may trigger increased antigen presentation and recognition by CD8^+^ T cell, leading to an enhanced T response. Importantly, we found that adoptive transfer of T cells from cabozantinib-treated mice bearing Renca tumors inhibited growth of Renca tumors in immunodeficient NPG mice.

Cabozantinib is a tyrosine kinase inhibitor that targets VEGFR2, MET and RET as well as KIT, AXL, and FLT3. All of these pathways have been found to be important for tumor survival, growth, metastasis and angiogenesis. Reducing angiogenesis by cabozantinib has been illustrated in previous study (6). In the current study, we found that depletion of either neutrophils or CD8^+^ T cell only partially abrogated the therapeutic effect of cabozantinib on RCC. These findings revealed the important role of neutrophils and T cells in cabozantinib-mediated anti-tumor effects and suggested that inducing neutrophils and T cells to infiltrate into the tumor bed and activate their anti-tumor activity is one of the mechanisms through which cabozantinib exerts its anti-tumor effects *in vivo*. It will be of interest to investigate whether cabozantinib treatment enhances infiltration and anti-tumor activity of neutrophils in patients with RCC in future studies.

## Conclusion

We revealed that the tyrosine kinase inhibitor cabozantinib inhibits the growth of murine renal cancer by activating both innate and adaptive immunity. These findings are of great significance for guiding the clinical use of cabozantinib and provide a good candidate for future combination therapy with T-cell therapies or other immunotherapies for RCC.

## Data Availability Statement

The original contributions presented in the study are included in the article/supplementary material. Further inquiries can be directed to the corresponding authors.

## Ethics Statement

The studies involving human participants were reviewed and approved by Ethics Committee of the Affiliated Hospital of Xuzhou Medical University. The patients/participants provided their written informed consent to participate in this study. The animal study was reviewed and approved by Institutional Ethical Care and Use Committee of Xuzhou Medical University.

## Author Contributions

QZ and JZ designed the project and experiments. HL, SS, ML, and GW performed experiments. HL interpreted the results of the experiments. QZ and HL wrote the manuscript. All authors contributed to the article and approved the submitted version.

## Funding

This work was supported by grants from the National Science Foundation of China (81773253), Jiangsu Province Social Development Projects (BE2018633), Youth Technology Innovation Team of Xuzhou Medical University (TD202003), Jiangsu Provincial Key Medical Discipline, the Project of Invigorating Health Care through Science, Technology and Education (ZDXKA2016014).

## Conflict of Interest

The authors declare that the research was conducted in the absence of any commercial or financial relationships that could be construed as a potential conflict of interest.
