# Supplementary material for: Tyrosine Kinase Inhibitor Cabozantinib Inhibits Murine Renal Cancer by Activating Innate and Adaptive Immunity
Source: Front Oncol. 2021 Apr 19;11:663517. doi: 10.3389/fonc.2021.663517 (PMC8089383; doi:10.3389/fonc.2021.663517)
Supplement: Supplementary file 1 [file DataSheet_1.docx]

Supplementary Material

**Supplementary Table 1.** Relationship between infiltrated neutrophils and clinicopathological

characteristics of individuals in two cohorts of ccRCC patients.

| **Variables** | **Validation cohort ( 307 cases )** | | | |
| --- | --- | --- | --- | --- |
|  | **Low (%)** | **High (%)** | ***P*** |  |
| **Age** |  |  |  |  |
| ≤56 years | 72 (48.6) | 76 (51.4) | 0.168 |  |
| >56 years | 64 (40.3) | 95 (59.7) |  |  |
| **Gender** |  |  |  |  |
| Male | 98 (48.0) | 106 (52.0) | 0.069 |  |
| Female | 38 (36.9) | 65 (63.1) |  |  |
| **Tumor size** |  |  |  |  |
| ≤7 cm | 106 (44.7) | 131 (55.3) | 0.891 |  |
| >7 cm | 30 (42.9) | 40 (57.1) |  |  |
| **pT status** |  |  |  |  |
| pT_1-_ pT_2_ | 108 (43.4) | 141 (56.6) | 0.558 |  |
| pT_3_ -pT_4_ | 28 (48.3) | 30 (51.7) |  |  |
| **pN status** |  |  |  |  |
| pN_0_ | 127 (45.2) | 154 (54.8) | 0.410 |  |
| pN_1_ –pN_3_ | 9 (34.6) | 17 (65.4) |  |  |
| **TNM stage** |  |  |  |  |
| I- II | 98 (44.3) | 123 (55.7) | 0.759 |  |
| III- IV | 25 (47.2) | 28 (52.8) |  |  |

^*^ Two sided Fisher’s exact tests.

Some cases were not available for the information in the validation cohort.

**Supplementary Table 2**. Univariate Cox regression analysis of neutrophil infiltration and clinicopathological variables predicting survival of renal cancer patients.

| Variables | Overall survival | | Disease free survival | |
| --- | --- | --- | --- | --- |
|  | HR (95%CI) | *P* | HR (95%CI) | *P* |
| Infiltrated neutrophils (Low *vs.* High) | 0.71(0.54–0.94) | 0.016 | 0.72 (0.53–0.97) | 0.031 |
| Age (≤56 *vs.* >56) | 1.07 (0.81–1.41) | 0.637 | 1.03 (0.76–1.39) | 0.840 |
| Tumor diameter (≤7 cm *vs.* >7 cm) | 1.67 (1.19–2.35) | 0.003 | 1.46 (0.98–2.18) | 0.063 |
| pT status (pT1/pT2 *vs.* pT3/pT4) | 1.51 (1.07–2.12) | 0.019 | 1.33 (0.89–1.96) | 0.157 |
| TNM stage (I–II *vs.* III–IV) | 1.64 (1.16–2.33) | 0.005 | 1.38 (0.93–2.06) | 0.111 |

Abbreviations: HR: hazard ratio; CI: confidence interval.

**Supplementary Table 3**. Multivariate Cox regression analysis models assessing the effects of covariates on OS and DSS in CRC patients.

| Variables | Overall survival | | Disease free survival | |
| --- | --- | --- | --- | --- |
|  | HR (95%CI) | *P* | HR (95%CI) | *P* |
| Infiltrated neutrophils (Low *vs.* High) | 0.61 (0.45–0.82) | 0.001 | 0.62 (0.45–0.85) | 0.003 |
| Age (≤56 *vs.* >56) | 0.92 (1.01–1.35) | 0.926 | 0.99 (0.73–1.35) | 0.936 |
| Tumor diameter (≤7 cm *vs.* >7 cm) | 1.43 (0.99–2.07) | 0.054 | 1.40 (0.92–2.12) | 0.115 |
| TNM stage (I–II *vs.* III–IV) | 1.72 (1.20–2.45) | 0.003 | 1.49 (0.99–2.24) | 0.055 |

Abbreviations: HR: hazard ratio; CI: confidence interval.

**Supplementary Table 4.** Relative expression levels of 25 chemokines in cabozantinib- or vehicle-treated tumors.

| Name | Vehicle | Cabozantinib | *P* |
| --- | --- | --- | --- |
| CCL21 | 1817 | 1856 | 0.7354 |
| CXCL13 | 1882 | 2191 | 0.1377 |
| CCL28/MEC | 1836 | 1867 | 0.8569 |
| CXCL16 | 2497 | 2193 | 0.0201 |
| CCL3/CCL4 | 2229 | 2235 | 0.9931 |
| CCL5 | 1362 | 1038 | 0.0284 |
| CCL6 | 41887 | 43798 | 0.6186 |
| C5/C5A | 11874 | 9820 | 0.4662 |
| CCL8/MCP-2 | 21664 | 49218 | 0.0008 |
| CCL12 | 26937 | 35159 | 0.0002 |
| CCL9/10 | 38217 | 36600 | 0.8234 |
| RARRES2 | 15777 | 19591 | 0.2175 |
| CCL28 | 8598 | 8561 | 0.9566 |
| CXCL1 | 7966 | 9425 | 0.3647 |
| CXCL9 | 3941 | 6151 | 0.0695 |
| CXCL10 | 3820 | 4952 | 0.2430 |
| CXCL12 | 5782 | 8095 | 0.0020 |
| CXCL11 | 2421 | 2519 | 0.7004 |
| CCL27 | 3737 | 3280 | 0.1078 |
| CCL11 | 2251 | 7969 | <0.0001 |
| CXCL2 | 5311 | 2743 | 0.2677 |
| CCL22 | 4287 | 4195 | 0.7953 |
| GCP-2 | 2804 | 3055 | 0.7667 |
| CX3CL1 | 2542 | 4407 | 0.0029 |

Chemokine expression levels are presented as arbitrary units measured by densitometry. (n=2, biological replicates). Mean densitometric values were calculated from duplicate blots (n=2).


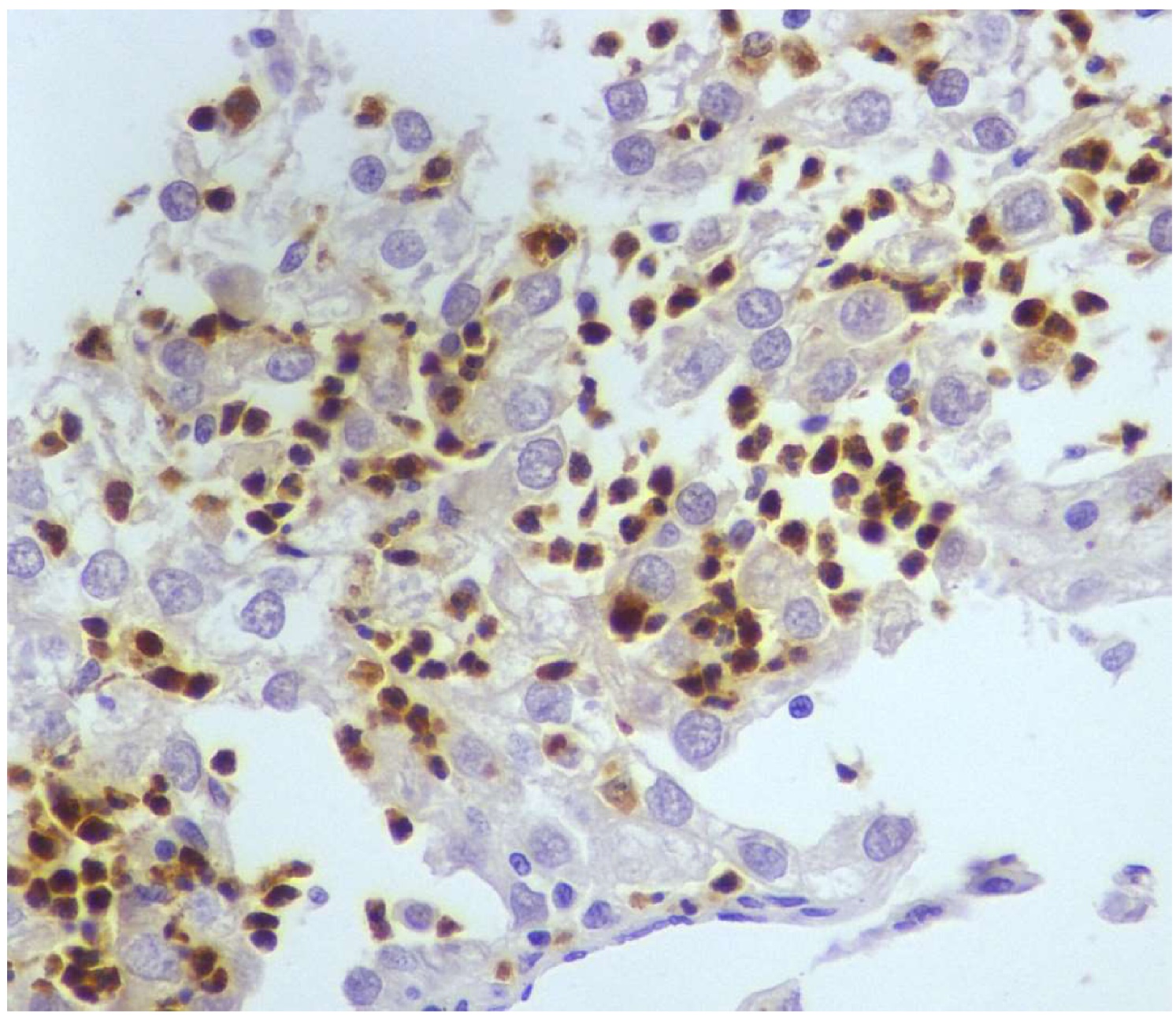


**Supplemental Figure 1. Representative IHC staining with MPO antibody shows segmented-nuclei neutrophils.** Magnification, X 600.


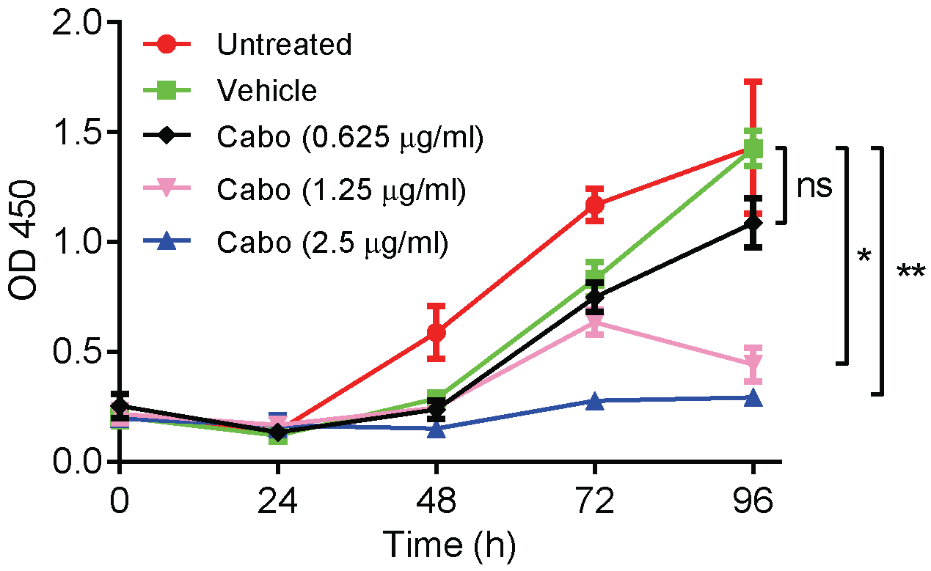


**Supplemental Figure 2. Proliferation inhibitory efficacy of cabozantinib in Renca cells.** Renca cells were treated with different concentrations of cabozantinib (0.625-2.5 μg/ml). Cell proliferation was assessed by CCK8 assay.


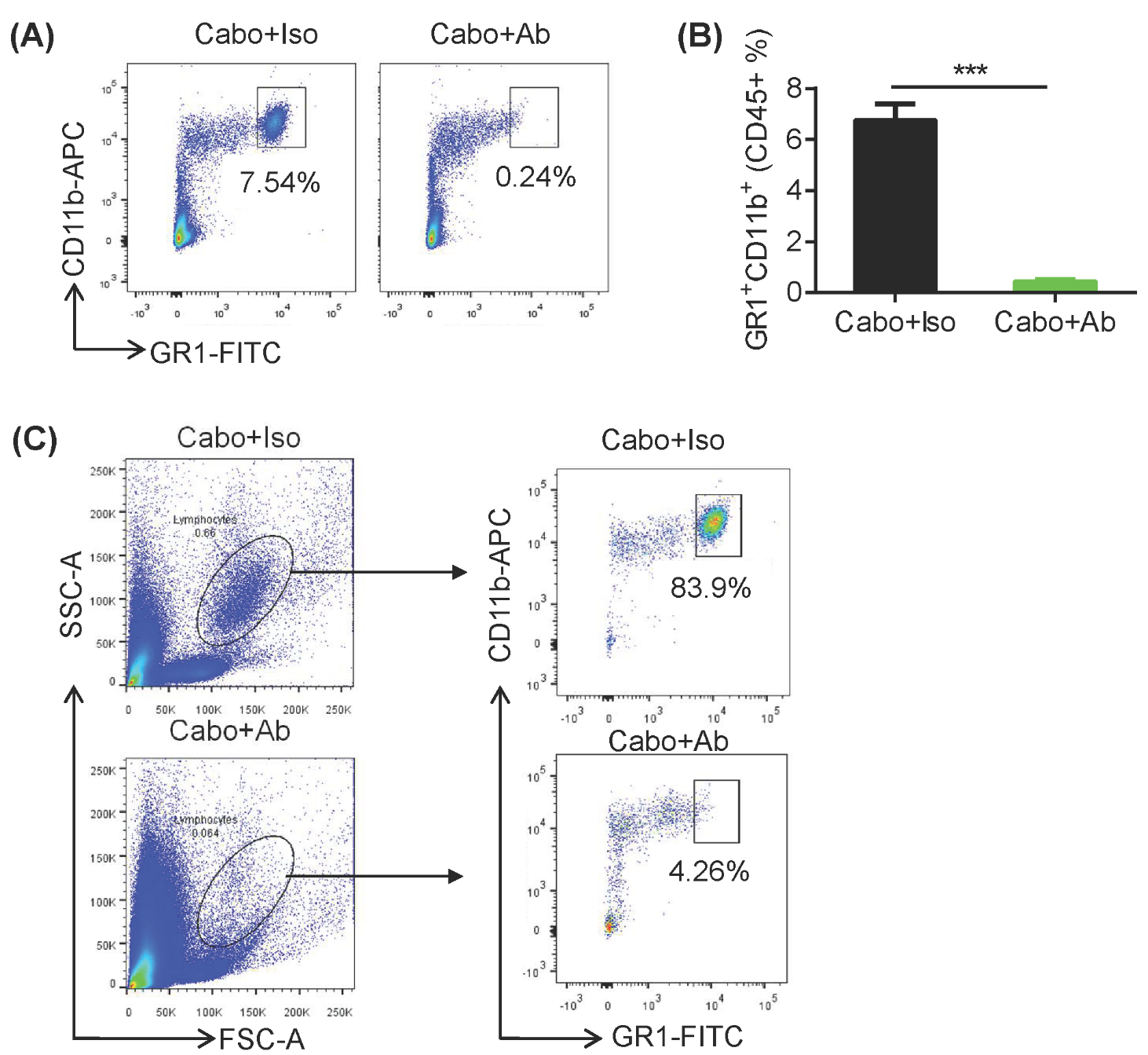


**Supplemental Figure 3. Analysis of neutrophil depletion effects.** Mice were treated as in Fig. 3C. (A-B) Representative FACS and statistical results of neutrophils through staining with anti-GR1 antibody (RB6-8C5). (C) FACS analysis through FSC/SSC gating strategy. Mice were treated with anti-Ly6G-depleting antibody (1A8). 24 hours after the first treatment, neutrophils in peripheral blood were detected by FACS. Representative data of three independent experiments are shown (cabozantinib + isotype，n=18; cabozantinib + anti-Ly6G-depleting antibody, n=18 in total) with mean values ± SEM. ****P*<0.001 by unpaired t-test.


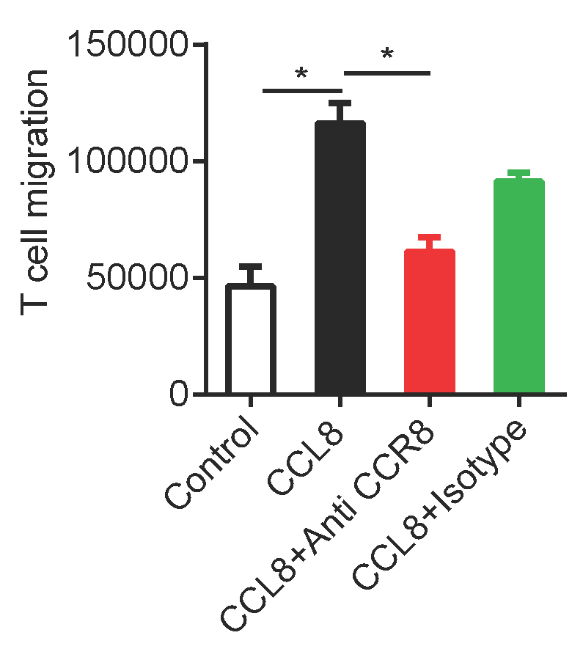


**Supplemental Figure 4.** **Analysis of chemotaxis of CCL8 on T cells.** T cells were isolated from splenocytes of Balb/c mice and blocked with antibodies against CCR8 or isotype control for 30 min, respectively. Untreated T cells or blocked T cells were added to the upper chambers of the transwells, respectively. Medium containing recombinant CCL8 was added to the lower chamber, respectively. After incubation for 6 h, the migratory cells were counted under the microscope. Representative data of two independent experiments are shown. n=3; data are shown as the mean values ± SEM. **P*<0.05, by unpaired t-test.
